# Hyperuricaemic *Urah*^*Plt2/Plt2*^ mice show altered T cell proliferation and defective tumor immunity after local immunotherapy with Poly I:C

**DOI:** 10.1371/journal.pone.0206827

**Published:** 2018-11-01

**Authors:** Camille Baey, Jianping Yang, Franca Ronchese, Jacquie L. Harper

**Affiliations:** Malaghan Institute of Medical Research, Wellington, New Zealand; University of Michigan Health System, UNITED STATES

## Abstract

Hyperuricaemia is associated with various metabolic dysfunctions including obesity, type 2 diabetes mellitus, hypertension and in general metabolic syndrome, which are all associated with increased risk of cancer. However, the direct association between elevated uricemia and cancer mortality still remains unclear. In this study, we used a mouse model of hyperuricemia, the *Urah*^*plt2/plt2*^ (PLT2) mouse, to investigate the effect of high uric acid levels on anti-tumor immune responses and tumor growth. In normo-uricaemic C57BL/6 mice injected with B16 melanomas, immunotherapy by treatment with Poly I:C at the tumor site delayed tumor growth compared to PBS treatment. In contrast, Poly I:C-treated hyper-uricaemic PLT2 mice were unable to delay tumor growth. Conventional and monocyte-derived dendritic cells in the tumor-draining lymph nodes (dLN) of C57BL/6 and PLT2 mice were similarly increased after Poly I:C immunotherapy, and expressed high levels of CD40 and CD86. CD8^+^ T cells in the tumor-dLN and tumor of both WT and PLT2 mice were also increased after Poly I:C immunotherapy, and were able to secrete increased IFNγ upon *in vitro* restimulation. Surprisingly, tumor-specific CD8^+^ T cells in dLN were less abundant in PLT2 mice compared to C57BL/6, but showed a greater ability to proliferate even in the absence of cognate antigen. These data suggest that hyperuricaemia may affect the functionality of CD8^+^ T cells *in vivo*, leading to dysregulated T cell proliferation and impaired anti-tumor activity.

## Introduction

Metabolic syndrome (MS) describes the metabolic dysfunction associated with obesity and increased risk of type 2 diabetes mellitus and cardiovascular diseases. The alarming increase in MS has become a major health concern internationally. These concerns arise not only from the life-threatening nature of the MS itself but are also due to the negative impact it has on other diseases including cancer [1–3]. Latterly, there is a growing body of epidemiological data that shows a strong link between MS and a number of different cancers in both men and women, ranging from colorectal [4, 5] through to prostate [6] and breast cancers [7, 8].

The multifactorial nature of MS makes it difficult to determine the contribution(s) of single, or groups of, components towards the negative impact of MS on cancer [5, 9]. Nevertheless, there is evidence of links between carcinogenesis and changes in glucose and/or lipid metabolism [10, 11], as well as chronic MS-related inflammation [12, 13].

One condition commonly observed with MS is chronic hyperuricaemia—uric acid build up resulting from the dysregulation of purine metabolism and/or uric acid clearance. Most commonly known for its role in the development of gout, epidemiological studies also link hyperuricaemia with carcinogenesis [14] where gout and hyperuricaemia are both associated with both risk of cancer and poor cancer prognosis [10, 15, 16].

*In vivo* experimental models of MS exhibit dysfunctional purine metabolism and elevated uric acid levels [17]. As in the clinical setting, the challenge of using these models to investigate the impact of purine metabolism in conditions like cancer is the presence of confounding factors such as obesity and diabetes. Previous work looking at the interference of purine metabolism in normal weight mice provides an opportunity to investigate the association between purine metabolism and cancer in the absence of these confounding factors. The *Urah*^*plt2/plt2*^ (PLT2) mice carrying a mutation of the *Urah* gene encoding mouse 5-hydroxyisourate hydrolase (HIU-H) show increased platelet numbers and hepatomegaly [18]. Both of these defects are rescued by expression of the intact form of the *Urah* gene, demonstrating the essential role of the *Urah*^*plt2/plt2*^ mutation in this phenotype. In addition, as predicted on the basis of the essential function of HIU-H in urate catabolism, the *Urah*^*plt2/plt2*^ mutation also results in perturbed purine metabolism and chronically increased uricemia without affecting body weight.

Effective anti-cancer therapies often exploit the immune system to boost anti-cancer responses by enhancing the inflammatory functions of key immune cells including antigen-presenting dendritic cells (DC) and CD8^+^ effector T cells (reviewed in [19, 20]). A number of studies identify the crystalline form of uric acid (monosodium urate-MSU) as an effective adjuvant that can boost a variety of immune responses, and has been shown to enhance the effectiveness of anti-cancer therapies in experimental models [21, 22].

Although elevated serum uric acid has been targeted in cancer in the context of Tumor Lysis Syndrome arising as a side effect of anti-tumor therapies [23], to date there has been no focus on how a pre-existing background of altered purine metabolism might impact on cancer treatment. In this study, we utilized the PLT2 model to investigate the impact of perturbed purine metabolism on both the tumorigenesis of B16 melanoma and the effectiveness of immune-mediated anti-tumor therapy. How altered purine metabolism affected classical components linked with successful anti-cancer immune responses was also investigated, with a focus on DC phenotypes and CD8^+^ T cell functions.

## Materials and methods

### Mice

All mice were bred at the Malaghan Institute of Medical Research Biomedical Research Unit and were matched for age and sex within experiments. C57BL/6J were originally from The Jackson Laboratory, Bar Harbor, ME. C57BL/6-background *Urah*^*plt2/plt2*^ (PLT2) mice were kindly provided by Dr. Warren Alexander, Walter and Eliza Hall Institute of Medical Research, Australia. CD45.1-congenic OT-I mice were obtained by crossing OT-I mice expressing a transgenic TCR specific for the ovalbumin peptide OVA_257-264_ bound to K^b^ (kindly provided by Prof. F. Carbone, Melbourne University, Australia) to B6.SJL-Ptrprca (CD45.1^+^) mice in house. TAP1 KO mice [24] on a C57BL/6 background were originally obtained from the Walter and Eliza Hall Institute of Medical Research. Animals were monitored daily during experiment and sacrificed by cervical dislocation. All experimental procedures were approved by the Victoria University of Wellington Animal Ethics Committee.

### Tumor cell lines and tumor challenge

The B16-F1 murine melanoma (American Type Culture Collection, ATCC) and B16.OVA melanoma expressing a truncated OVA protein [25] were maintained in complete IMDM as described [26]. Extended *in vitro* passaging was avoided for all cell lines. For tumor challenge, cells were washed 3 times in PBS and 2x10^5^ tumor cells were suspended in 100μl of PBS and injected *s*.*c*. into the flank of mice. Groups of 5 mice were used in most experiments unless otherwise specified. Tumor size and survival were calculated as described [26] and mice culled when tumor reached 150mm^2^.

### Immunotherapy treatment

Mice were treated with 50μg Poly I:C (InvivoGen) or 20μg LPS (Sigma) in a total volume of 100μl PBS injected *s*.*c* around the palpable tumor, at the times indicated. Control mice received 100μl vehicle alone (PBS, Invitrogen). Non-bearing tumor mice were treated *s*.*c*. with Poly I:C (50μg) for 24h to assess DC maturation state.

### Analysis of uric acid levels

C57BL/6J or PLT2 mice were bled by cheek puncture and blood was collected in microtubes containing 5μl of EDTA. After 20 min of centrifugation at 14 000 rpm, plasma was stored in micro tubes at -70°C. Uric acid levels were measured using Uric Acid Assay Kit (Cayman Chemical).

### Flow cytometry

LN and tumors were digested using DNase I and Liberase TL (Roche) and passed through 70μm cells strainers (BD Biosciences) to obtain single cell suspensions. Cells were then resuspended in FACS buffer (PBS with 10mM EDTA, Sigma; 2% FBS, Gibco; and 0.01% NaN_3_, Sigma) and blocked with anti-mouse CD16/32 (2.4G2) before staining with combinations of the following antibodies: CD45 (30-F11), CD45.1 (A20), CD3 (145-2C11), CD8α (53–6.7), CD11b (M1/70) and Ly6G (1A8), all from BD Biosciences; CD45 (30-F11), MHCII (M5/114.15.2), Ly6C (HK1.4) and NK1.1 (PK136) from Biolegend; CD45R (RA3-6B2), CD3 (145-2C11), and F4/80 (BM8) from eBioscience and Ly6B (7/4) from AbD Serotec. CD8 (2.43) and CD11c (N418) were affinity purified from hybridoma supernatant. Dead cells were excluded as staining positive for 4’,6-diamidino-2-phenylindole (DAPI) or Live/Dead Fixable blue (Invitrogen). For intracellular cytokine staining of T cells, cell suspensions were incubated for 6h at 37°C in 1μg/ml Golgi Stop (BD Biosciences) and human recombinant IL-2 (in house, 10^2^U/ml) with anti-CD3 (5μg/ml) and anti-CD28 (2μg/ml) restimulation. Cells were first stained for surface markers, followed by intracellular staining with anti-IFNγ (BD Biosciences) antibody or isotype control using BD Cytofix/Cytoperm kit (BD Biosciences). Flow cytometry data was acquired using a BD LSRII or a BD LSRFortessa (Beckton Dickinson) and data were analyzed using FlowJo version 9.9. Representative gating is shown in S1 Fig.

### Assessment of NK cell cytotoxicity *in vivo*

Mice were injected i.v. with 100μg Poly I:C or PBS as a control. Thirty-six hours later, mice were injected with a 1:1 mixture of CFSE-labelled C57BL/6 and CTO-labelled TAP KO target spleen cells, as described [27]. Mice were euthanized 6h later and the proportion of CFSE and CTO-labelled cells in spleen was determined by flow cytometry. Percent killing was calculated according to the following formula: 100-[(CTO target / CFSE target in test sample)/(CTO target / CFSE target before injection)]*100.

### *In vivo* T cell proliferation

CD8^+^ T cells from spleens and LNs of OT-I mice were positively selected using Mouse CD8 FlowComp Dynabeads (Invitrogen) and labeled with CFSE, as described [28]. Flow cytometry analysis confirmed >90% purity as assessed by positive staining with CD8α, Vα2 and Vβ5 antibodies. 1–2 x10^6^ cells were injected *i*.*v* into C57BL/6J or PLT2 mice bearing established day 8 B16.OVA tumors. Mice were treated with Poly I:C the following day and OT-I proliferation was assessed in tumor-dLN 3 days later by evaluating CFSE dilution.

### Analysis of serum cytokines

Peripheral blood was collected 3h after the first peritumoral treatment and left to clot overnight (16h) at 4°C. Serum was separated by centrifugation, and cytokines were measured using a custom pre-mixed Millipore Mouse Cytokine Magnetic Bead Panel kit and a Bio-Plex reader (Bio-Rad), according to the manufacturer’s instructions.

### Statistics

Statistical analyses were performed using Prism 7.0 software (GraphPad). Means ± SEM are shown in all graphs. Tumor survival data were analyzed using the log-rank test with the Bonferroni correction. In all other cases, data were analyzed using two-way ANOVA with Bonferroni correction unless otherwise specified.

## Results

### Poly I:C tumor immunotherapy is ineffective in PLT2 mice

**Fig 1:**
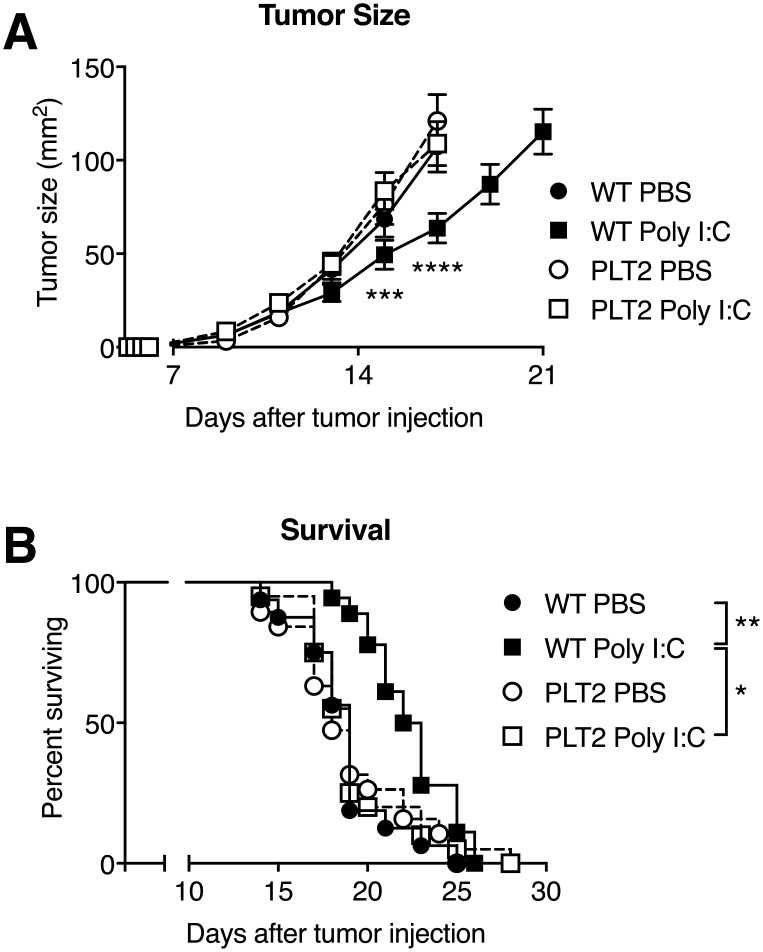
Poly I:C immunotherapy is ineffective in hyperuricaemic PLT2 mice. C57BL/6 (WT) and PLT2 mice were challenged with B16 tumor cells and given peri-tumoral injections of PBS or Poly I:C on days 9, 11, 13 and 15. (**A**) Tumor growth. Graph shows mean tumor size±SEM in groups of 15 mice pooled from three independent experiments. Statistical analysis was by 2-way ANOVA with Tukey’s correction, the p values shown in Figure refer to the comparison between WT Poly I:C and PLT2 Poly I:C. ***p<0.001, ****p<0.0001. (**B**) Kaplan-Meier survival curves. Data are pooled from four independent experiments, n = 20 mice/group. Statistical analysis was by log-rank test with Bonferroni correction; *p<0.05, **p<0.01.

We tested the impact of the point mutation in the gene encoding for HIU-H on the efficacy of tumor immunotherapy in C57BL/6 wild-type (WT) and PLT2 mice challenged with B16-F1 melanoma. Tumor growth and survival were comparable for WT and PLT2 PBS-treated control mice but, where Poly I:C treatment delayed tumor growth and extended survival in WT mice, Poly I:C treatment had no effect in tumor-bearing PLT2 mice (Fig 1A and 1B).

**Fig 2:**
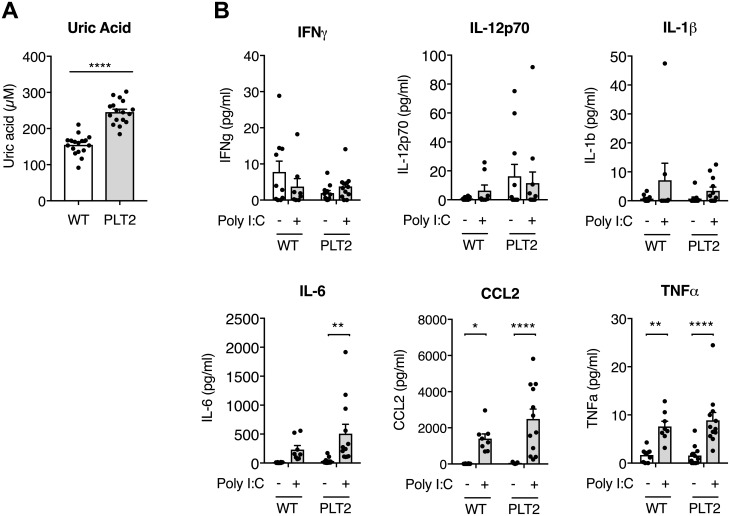
Poly I:C immunotherapy induces serum cytokine responses in both C57BL/6 and PLT2 mice. (**A**) Uric acid levels in untreated C57BL/6 (WT) and PLT2 mice. Mice were gender and age-matched between groups. The bar graph shows mean±SEM for 11 females and 9 males/group, ranging between 9 and 13 weeks of age. Each dot corresponds to one mouse. Statistical analysis was by Mann-Whitney test with ranks comparison, ****p<0.0001. (**B**) C57BL/6 and PLT2 mice were challenged with B16 tumors and treated with PBS or Poly I:C on day 9. Serum samples were collected three hours later. Cytokine levels were measured by a multiplex bead assay. Bar graphs show the mean+SEM of pooled data from two independent experiments for a total of 8–12 mice/group; each dot corresponds to one mouse. Statistical analysis was by two-way ANOVA with Bonferroni’s post-test. *p<0.05, **p<0.01, ****p<0.0001.

Consistent with impaired HIU-H function, PLT2 mice exhibited elevated serum uric acid levels compared with sex and age-matched WT mice (Fig 2A). As uric acid has been linked to elevated inflammatory responses, we also compared the effect of Poly I:C treatment on serum cytokine levels in tumor-bearing WT and PLT2 mice. Although Poly I:C treatment did raise the levels of IL-6, MCP-1 and TNFα, there were no significant differences between WT and PLT2 mice (Fig 2B).

### Poly I:C-treatment induces DC activation in PLT2 mice

**Fig 3:**
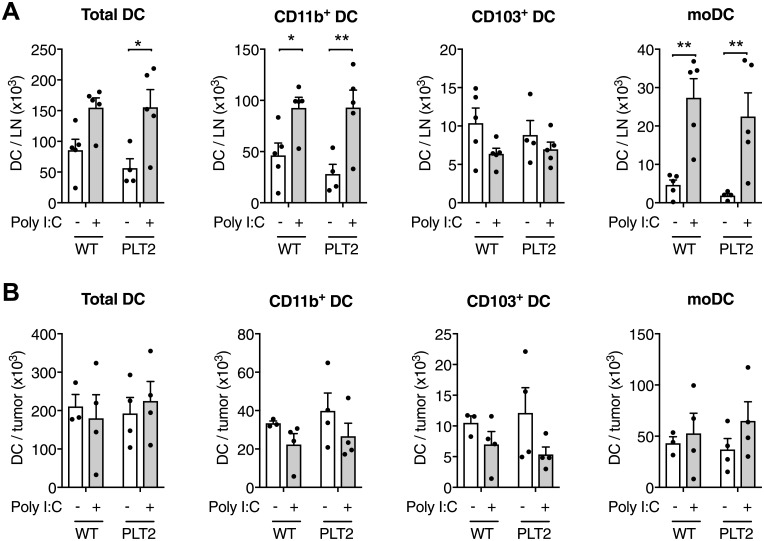
Poly I:C immunotherapy similarly increases DC numbers in the tumor-dLN and tumor of C57BL/6 and PLT2 mice. C57BL/6 (WT) and PLT2 mice were challenged with B16 tumor cells and treated with peri-tumoral injections of PBS or Poly I:C on days 9, 11, 13 and 15. On day 17 the tumors and tumor-dLN were removed and analyzed by flow cytometry using the gating strategy shown in S1 Fig. (**A**) Numbers of total DC (CD11c^+^MHCII^+^), CD11b^+^ DC (CD11c^+^MHCII^+^CD11b^+^ CD103^-^CD8^-^Ly6C^-^Ly6B^-^), CD103^+^ DC (CD11c^+^MHCII^+^CD11b^-^) and monocyte-derived DC (CD11c^+^MHCII^+^CD11b^+^Ly6C^+^Ly6B^+^) per LN. (**B**) Numbers of the same DC subsets in tumors. Bar graphs show mean+SEM for one of three independent experiments, each with 3–5 mice/group, that gave similar results. Each dot corresponds to one mouse. Statistical analysis was by two-way ANOVA with Bonferroni’s post-test, *p<0.05, **p<0.01.

We examined DC populations in the tumor-draining LN (dLN) and tumors of WT and PLT2 mice. As shown in Fig 3A and S2 Fig, Poly I:C treatment caused a similar increase in the number of total CD11c^+^ MHCII^+^ DC in the dLN of WT and PLT2 mice. Further DC analysis in WT and PLT2 mice showed no differences in the numbers of CD11b^+^, CD103^+^, CD8^+^ or monocyte-derived DC (moDC) subpopulations. Analysis of the same DC populations in tumors also showed no differences between WT and PLT2 mice (Fig 3B).

We have reported previously that Poly I:C tumor immunotherapy is associated with increased moDC in the dLN of tumor-bearing mice [21]. As PLT2 and WT mice exhibited comparable changes in DC populations following Poly I:C treatment, we examined phenotypic markers (CD86, CD40, MHCII) linked to DC immune function that might be differentially affected in Poly I:C-treated PLT2 mice. LPS treatment was also included as an immunotherapy negative control [21]. As expected, Poly I:C treatment elevated the number of total DC, CD11b^+^ DC, CD103^+^ DC and moDC in the dLN (S3A Fig) while LPS was ineffective. Flow cytometry analysis of the CD11b^+^ and CD103^+^ DC and moDC populations showed similar CD86 and CD40 expression on DC from WT and PLT2 mice (S3B Fig), suggesting similar DC responses to immunotherapy.

### Poly I:C immunotherapy primes CD8^+^IFNγ^+^ T cells in PLT2 mice

**Fig 4:**
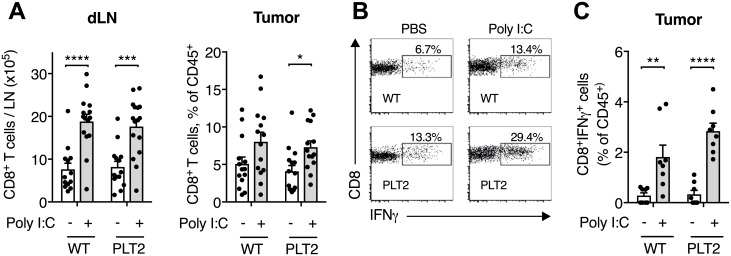
Poly I:C immunotherapy increases the proportion of intratumoral CD8^+^IFNγ^+^ T cells in C57BL/6 and PLT2 mice. C57BL/6 (WT) and PLT2 mice were challenged with B16 tumor cells and treated with peri-tumoral injections of PBS or Poly I:C on day 9, 11, 13 and 15. On day 17, tumor-dLN and tumors were removed for flow cytometry analysis. (**A**) Total numbers of CD8^+^ T cells in dLN and tumors. (**B**) Representative flow plots showing the proportion of IFNγ^+^ cells in the intratumoral CD8^+^ T cell population as determined by intracellular cytokine staining. (**C**) Proportions of CD8^+^IFNγ^+^ cells in tumors, expressed as percentage of the CD45^+^ population. Bar graphs show mean+SEM for pooled data from 2–5 independent experiments, each with 4–5 mice/group, that gave similar results. Each dot refers to one mouse. Statistical analysis was by two-way ANOVA with Bonferroni’s post-test. *p<0.05, **p<0.01, ***p<0.001, ****p<0.0001.

We have shown previously that the anti-tumor effect of Poly I:C requires CD8^+^ T cells [21], and that IFNγ production by CD8^+^ T cells is essential for anti-tumor activity [29]. As shown in Fig 4A, WT and PLT2 mice showed similar increases in CD8^+^ T cells in both tumor-dLN and tumors. In addition, the proportion of CD8^+^ T cells producing IFNγ in tumors from WT and PLT2 mice were both increased after Poly I:C immunotherapy compared to control (Fig 4B). The proportions of CD8^+^IFNγ^+^ T cells in the tumor immune infiltrate were also increased in Poly I:C-treated WT and PLT2 mice (Fig 4C).

Besides promoting DC activation and CD8^+^ T cell responses, Poly I:C is a strong inducer of NK cell anti-tumor activity [30]. NK cell numbers in the tumor-dLN of WT and PLT2 mice were increased after Poly I:C immunotherapy, and this increase was more marked in WT compared to PLT2 (S4A Fig). Conversely, a trend to increased proportions of NK cells after Poly I:C immunotherapy was apparent in tumors and reached statistical significance only in PLT2 mice (S4A Fig) In addition, Poly I:C treatment elicited similar *in vivo* NK cell-mediated cytotoxic activity in non tumor-bearing mice (S4B Fig).

### The proliferation of tumor-specific CD8^+^ T cells is increased in PLT2 mice

**Fig 5:**
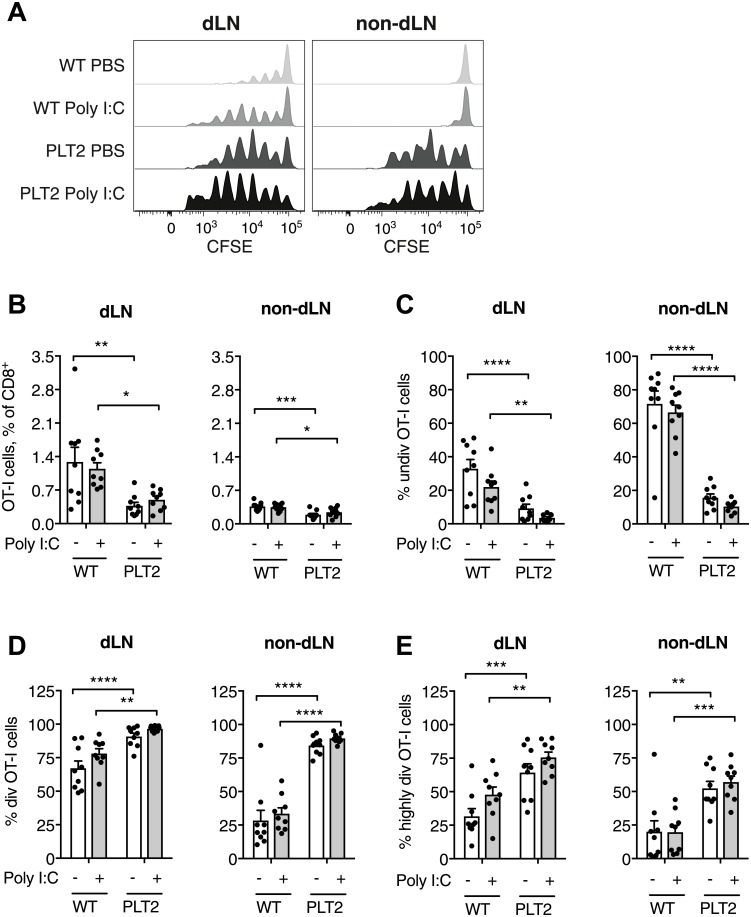
Tumor-specific CD8^+^ T cells proliferate more vigorously in PLT2 than C57BL/6 mice irrespective of Poly I:C immunotherapy. C57BL/6 (WT) and PLT2 mice were challenged with B16.OVA tumor cells. CFSE-labelled OT-I T cells were adoptively transferred into tumor-bearing mice on day 8. Mice were treated with peri-tumoral injections of Poly I:C or PBS on day 9, and tumor-dLN were removed on day 12 for flow cytometry analysis. (**A**) Representative histograms of OT-I T cells in tumor-draining and contralateral LN. OT-I T cells were identified as CD8^+^CD45.1^+^. (**B**) Proportion of OT-I T cells in the CD8^+^ T cell population in draining and non-dLN. (**C-E**) Proportions of undivided, divided, and highly divided (4 or more divisions) OT-I T cells in draining and non-draining LN. Bar graphs show mean+SEM for pooled data from two independent experiments each with 4–5 mice/group. Each dot refers to one mouse. Statistical analysis was by two-way ANOVA with Bonferroni’s post-test. *p<0.05, **p<0.01, ***p<0.001, ****p<0.0001.

To investigate further the antigen-specific T cell response to tumor antigen, CSFE-labelled OVA-specific OT-I T cells were adoptively transferred into mice bearing B16-OVA tumors, and the effect of Poly I:C treatment on OT-I T cell proliferation in the dLN of WT and PLT2 mice was evaluated (Fig 5A).

As shown in Fig 5B there was a greater proportion of OT-I T cells in the CD8^+^ T cell population in the dLN compared to the non-dLN of tumor-bearing mice, with a higher proportion of OT-I T cells in the WT compared to PLT2 mice. Poly I:C treatment did not affect the proportion of OT-I T cells in the total CD8^+^ T cell population. Analysis of OT-I T cell proliferation by CFSE dilution showed more proliferation of OT-I T cells in the dLN and non-dLN of PLT2 mice than in WT mice (Fig 5A and 5C–5E). There were significantly fewer undivided OT-I T cells in PLT2 compared to WT mice in both the dLN and non-dLN of tumor-bearing mice (Fig 5A and 5C). There were also fewer undivided cells in the dLN compared to the non-dLN in both PLT2 and WT mice. Moreover, there was a trend of decreased undivided cells with Poly I:C treatment in both PLT2 and WT mice.

Consistent with the pattern for undivided OT-I T cells, we observed a greater proportion of divided and highly divided OT-I T cells in the dLN and non-dLN of tumor-bearing PLT2 mice compared to WT mice (Fig 5A, 5D and 5E). Whereas Poly I:C treatment had little effect on OT-I T cells in non-dLN, there was a trend towards more divided and highly divided OT-I T cells in the dLN of both PLT2 and WT mice, with the greatest impact on OT-I T cells in WT mice.

## Discussion

There is increasing evidence of MS and hyperuricaemia contributing to poor outcomes in cancer [1, 2, 9, 14]. In this study we used PLT2 mice that offer the benefit of stable hyperuricemia without the concomitant effects of drug-induced hyperuricemia models [31]. We found that dysfunctional purine metabolism caused elevated serum uric acid levels in PLT2 mice and negatively impacted on the effectiveness of Poly I:C immunotherapy to delay tumor growth in a subcutaneous model of B16 melanoma.

Clinical studies indicate that MS and hyperuricaemia may increase the incidence of some cancers such as colorectal cancer [4, 10]. PLT2 mice also show a predisposition to developing hepatocarcinoma over time [18]. In comparison, our findings showed growth of B16 tumors to be similar in both control WT and PLT2 mice. It is possible that the more acute nature of the B16 tumor model overrides the long-term effect of chronic purine metabolic dysfunction on tumor development and growth observed in other studies. The type of cancer as well as the site of tumor induction, skin versus liver, may also play a role.

Our data identify that disruption of purine metabolism alters the efficacy of the anti-tumor immunotherapy Poly I:C. It is postulated that elevated uric acid levels is a surrogate indicator of an inflammatory environment that favors tumorigenesis and enables tumor growth [14, 32, 33]. We did not observe any significant differences in the inflammatory cytokine profiles in the blood of tumor-bearing WT and PLT2 mice regardless of whether they received treatment with the anti-tumor agent Poly I:C. Serum levels of type I IFN were not measured in these experiments, but the similar DC activation profile in C57BL/6 and PLT2 mice, which is IFN-I-dependent [34], suggests no major defects in the production of this cytokine. As such it does not appear that differences in classical inflammatory cytokines contribute to the observed loss of anti-tumor activity in the PLT2 mice.

Antigen presenting cells, most commonly DC, are widely recognized as playing a pivotal role in raising anti-tumor responses in the treatment of cancer. Previous work on the adjuvant effects of crystalline monosodium urate (MSU) and mycobacteria in boosting cancer immunotherapy also points towards monocyte derived DC in orchestrating beneficial anti-tumor activity [21]. It is also proposed that soluble uric acid has the ability to enhance DC function [35, 36]. Our data showed no such enhanced activation of DC populations in hyperuricaemic PLT2 mice compared with WT in the steady-state, and no increased response to treatment with Poly I:C or LPS.

The expansion and activation of NK cells and antigen-specific IFNγ-producing CD8^+^ T cells are common targets for effective anti-tumor activity [19, 20]. In this study WT and PLT2 mice exhibited similar profiles for NK cells and for total CD8^+^ T cells as well as CD8^+^IFNγ^+^ T cells, indicating that the NK and CD8^+^ T cell phenotype might not be involved in the loss of anti-tumor activity in the PLT2 mice. However, closer inspection of the antigen-specific response indicated a significantly smaller proportion of antigen-specific CD8^+^ T cells present in PLT2 mice.

It has been reported that uric acid plays a key role in boosting CD8^+^ T cell responses in transplant and diabetes models including enhanced CD8^+^ T cell proliferation [35, 37]. It is therefore notable that PLT2 mice exhibited a greater proportion of proliferating antigen-specific OT-I T cells in both draining and non-draining LNs, before and after Poly I:C treatment, compared to WT mice without translating into better anti-tumor activity. The relationship between the increased proliferation of tumor-specific CD8^+^ T cells and their decreased proportion in the tumor-dLN of PLT2 mice remains unclear and may suggest that these highly divided cells were not surviving well in PLT2 hosts. It is also possible that this high background proliferation in the PLT2 mice might limit the ability of the Poly I:C treatment to further expand the antigen specific CD8^+^ T cell population sufficiently to raise an effective anti-tumor immune response.

Our findings introduce the concept that chronic dysfunctional purine metabolism and hyperuricaemia could in some cases compromise anticancer therapy. This may be particularly relevant in situations where cancer treatment results in extensive tissue/cell death leading to highly elevated uric acid levels. A number of urate-lowering therapies are already used in cancer treatment—primarily to control Tumor Lysis Syndrome. The potential for managing purine metabolism and uric acid as a means to improve anti-cancer therapy warrants further investigation.

## Supporting information

S1 Fig Gating of DC subsets. Flow cytometry gating used to define DC subsets in LN. (**A**) Conventional DC (cDC) were CD11c^+^MHCII^+^; CD11b^+^ DC were CD11c^+^MHCII^+^CD11b^+^ CD103^-^CD8^-^Ly6C^-^Ly6B^-^; CD8^+^ DC were CD11c^+^MHCII^+^CD11b^-^; CD103^+^ DC were CD11c^+^MHCII^+^CD11b^-^. (**B**) monocyte-derived DC (moDC) were CD11c^+^MHCII^+^Ly6C^+^Ly6B^+^ and expressed high CD11b (not shown).
(EPS)

S2 Fig Poly I:C immunotherapy increases DC numbers in the tumor-dLN of C57BL/6 and PLT2 mice. C57BL/6 (WT) and PLT2 mice were challenged with B16 tumor cells and treated with peri-tumoral injections of PBS or Poly I:C on days 9, 11, 13 and 15. On day 17 the tumor-dLN were removed and analyzed by flow cytometry. DC gating was as shown in S1 Fig. Numbers of total “conventional” DC, CD11b^+^ DC, CD8^+^ DC and monocyte-derived DC per LN. Bar graphs show mean and SEM for two combined experiments, each with 3–5 mice/group. Each dot corresponds to one mouse. Statistical analysis was by two-way ANOVA with Bonferroni’s post-test, *p<0.05, **p<0.01, ***p<0.001.
(EPS)

S3 Fig Effect of Poly I:C or LPS treatment on DC numbers and surface marker expression in PLT2 and WT mice. C57BL/6 (WT) and PLT2 mice were injected with PBS, LPS or poly I:C into the flank, and dLN were harvested 24h later for flow cytometry analysis. (**A**) Number of total DC, CD11b^+^ DC, CD103^+^ DC and moDC per LN. DC subsets were identified as in Fig 3. Data are pooled from three independent experiments, each with 3–4 mice/group, that gave similar results. Bar graphs show mean+SEM, each dot corresponds to one mouse. Statistical analysis was by two-way ANOVA with Bonferroni’s post-test; ***p<0.001, ****p<0.0001. (**B**) Surface expression of the activation markers CD40 and CD86 on the indicated DC subsets; representative samples from one experiment are shown.
(EPS)

S4 Fig Poly I:C immunotherapy increases the frequency of NK cells in the tumor-dLN of WT and PLT2 mice, and their cytotoxic activity. (**A**): Mice were treated with PBS or Poly I:C at the tumor site and euthanized after 4 treatments. NK cell numbers in tumor-dLN, and their frequencies in tumors, were calculated using flow cytometry. Data are pooled from three independent experiments, each with 3–5 mice per group. (**B**): Mice were treated intravenously with PBS or Poly I:C. Thirty-six hours later, mice were injected with a mixture of TAP KO and WT labeled splenocytes, and the relative proportion of TAP KO cells compared to WT was assessed 6h later to estimate killing. Data are pooled from two independent experiments each with three mice/group. Bar graphs show mean+SEM, each dot corresponds to one mouse. Statistical analysis was by two-way ANOVA with Bonferroni’s post-test; *p<0.05, **p<0.01, ****p<0.0001.
(EPS)
